# Pemigatinib treatment for intrahepatic cholangiocarcinoma with FGFR2 fusion detected by a liquid comprehensive genomic profiling test

**DOI:** 10.1002/ccr3.7664

**Published:** 2023-07-05

**Authors:** Shun Ishido, Nobuharu Tamaki, Kento Inada, Jun Itakura, Yuka Takahashi, Naoki Uchihara, Keito Suzuki, Yuki Tanaka, Haruka Miyamoto, Michiko Yamada, Hiroaki Matsumoto, Tsubasa Nobusawa, Taisei Keitoku, Kenta Takaura, Shohei Tanaka, Chiaki Maeyashiki, Yutaka Yasui, Kaoru Tsuchiya, Hiroyuki Nakanishi, Masayuki Kurosaki, Namiki Izumi

**Affiliations:** ^1^ Department of Gastroenterology and Hepatology Musashino Red Cross Hospital Tokyo Japan

**Keywords:** comprehensive genomic profiling, fibroblast growth factor receptor 2, intrahepatic cholangiocarcinoma, pemigatinib

## Abstract

The liquid CGP was useful for detecting FGFR2 fusion and the patient experienced typical side effects (nail disorders, hyperphosphatemia, and taste disorders) of pemigatinib that required treatment.

## Graphical abstract

In a patient with Stage IVB intrahepatic cholangiocarcinoma with FGFR2 fusion, typical pemigatinib side effects required treatment, but long‐term survival could be achieved by multidisciplinary management of side effects.

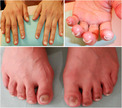

## 1 INTRODUCTION

Liver cancer has become the second and sixth most common cause of cancer‐associated deaths in men and women.^1^ Hepatocellular carcinoma (HCC) accounts for 75%–85% of patients with liver cancer and is a major health burden worldwide.^2^, ^3^ Intrahepatic cholangiocarcinoma (ICC) is a malignant neoplasm occurring in the epithelium of the biliary tract and the second most common primary liver cancer following HCC, accounting for approximately 15% of primary liver cancers and 3% of gastrointestinal tumors.^4^, ^5^ As ICC is often asymptomatic in its early stages and detected in advanced stages, curative therapy cannot be identified in many patients with ICC. Chemotherapy is the primary treatment for patients with advanced stage with the combination of gemcitabine and cisplatin (GC therapy); the combination of gemcitabine and S‐1; and the triple combination of gemcitabine, cisplatin, and S‐1 as the well‐established first‐line treatment options for unresectable ICC.^6^, ^7^, ^8^ However, no effective second‐line chemotherapy has been established due to insufficient clinical benefits.^9^, ^10^

Recently, comprehensive genomic profiling (CGP) has been approved and used in clinical practice. It can detect genetic alterations, may verify alternative treatment options, and achieve personalized treatment by inhibiting specific molecular drivers in critical signaling pathways.^11^ Fibroblast growth factor receptor 2 (FGFR2) fusion is found in ICC and occurs in up to approximately 10%–20% of patients.^4^ Pemigatinib is an FGFR1‐, FGFR2‐, and FGFR3‐selective inhibitor and is currently approved for the treatment of previously treated unresectable ICC with FGFR2 fusion.^12^ However, reports on using pemigatinib for the treatment of ICC are limited. Herein, we reported the case of a 53‐year‐old woman with stage IVB ICC who was treated with pemigatinib. In this case, there are two points worth mentioning: the liquid CGP was useful for detecting FGFR2 fusion and the patient experienced typical side effects of pemigatinib that required treatment.

## 2 CASE REPORT

**FIGURE 1:**
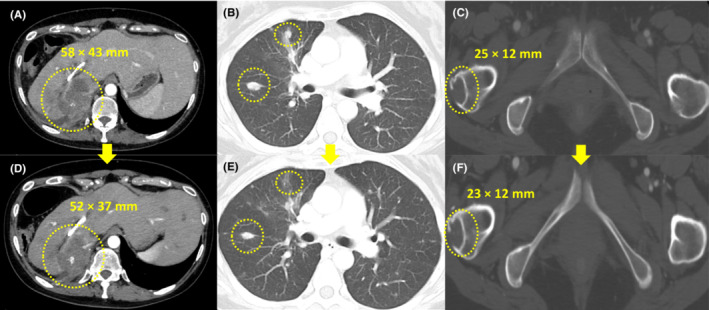
Two months after initiating pemigatinib. Computed tomography scan showing the primary tumor in the intrahepatic region, lung, and bone metastases before pemigatinib treatment (A–C). Two months after initiating pemigatinib (C–E), all lesions were determined to be stable disease (RECIST version1.1) with a reduced length diameter within 20%. RECIST, Response Evaluation Criteria in Solid Tumors.

**FIGURE 2:**
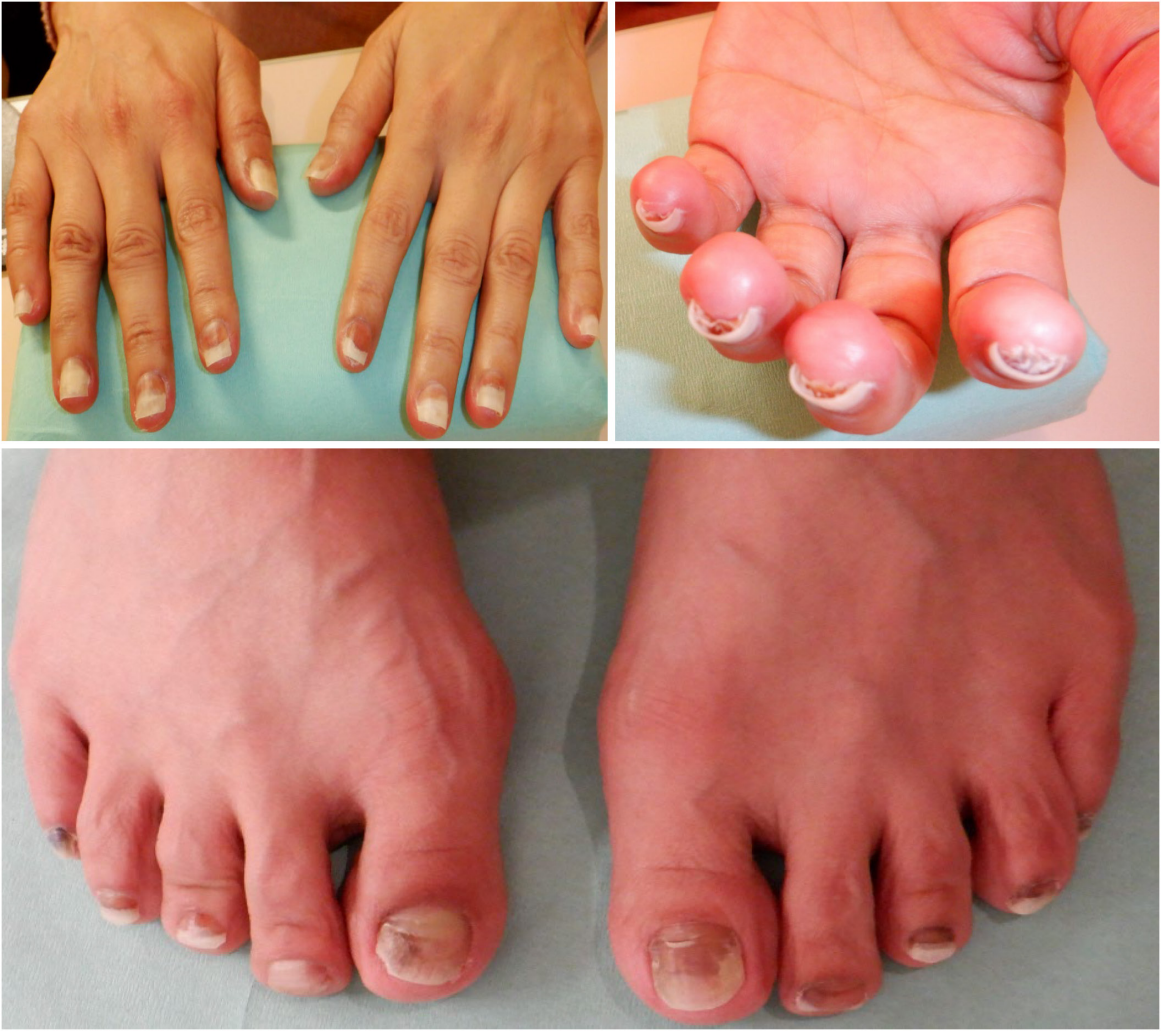
Nail disorders by pemigatinib.

**FIGURE 3:**
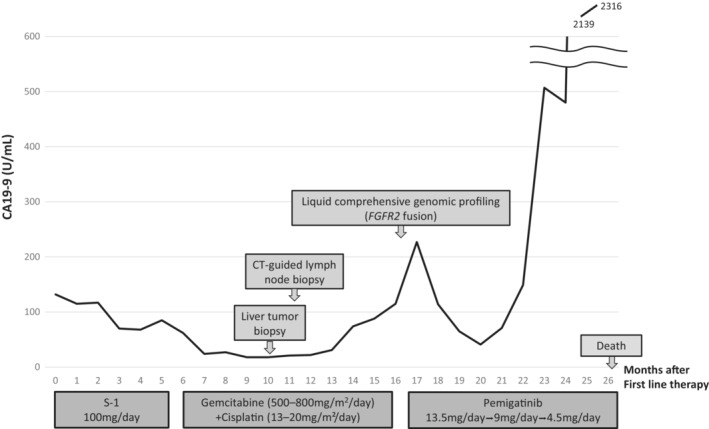
Treatment progression. CA19‐9, carbohydrate antigen 19–9; and CT, computed tomography; FGFR2, fibroblast growth factor receptor 2.

A 53‐year‐old woman presented to the hospital with a chief complaint of abdominal pain. Contrast‐enhanced computed tomography (CT) revealed a large tumor on the right lobe of the liver, multiple lung tumors, multiple enlarged lymph nodes, and peritoneal dissemination. A liver tumor biopsy was performed, which confirmed ICC. Therefore, the patient was diagnosed with unresectable ICC (cT4N1M1, cStage IVB). The patient had a history of gastric ulcers but without a family history of cancer, medication maintenance, and allergies. Due to extensive metastasis and declining performance status, the patient was treated with S‐1 (100 mg/day) as the first‐line treatment. Progressive disease (PD) due to increased pulmonary metastasis and peritoneal dissemination by Response Evaluation Criteria in Solid Tumors (RECIST) version 1.1 was observed after 5 months of S‐1 treatment, and gemcitabine of 500–800 mg/m^2^/day + cisplatin of 13–20 mg/m^2^/day (GC) therapy was started as the second‐line treatment. GC therapy was administered for 10 months; however, PD due to increased bone metastais and peritoneal dissemination occurred. Therefore, CGP was performed to identify treatment options. The specimen from the initial liver tumor biopsy performed to diagnose ICC was insufficient for CGP submission. Thus, liver tumor biopsy and CT‐guided lymph node biopsy were performed to inspect CGP; however, a viable specimen could not be obtained. Therefore, liquid CGP (FoundationOne® Liquid CDx, Foundation Medicine) was performed, and FGFR2 fusion was detected. The results revealed that this patient could use pemigatinib (13.5 mg/day) as the third‐line treatment. Two months after initiating pemigatinib, a CT scan showed controlled intrahepatic lesions and other distant metastases, and the state of the cancer was considered a stable disease based on RECIST (version 1.1) (Figure 1). However, specific side effects (nail disorders, hyperphosphatemia, and taste disorders) should be addressed after initiating pemigatinib. Because nail disorders were classified as Grade 3 (Criteria for adverse events version 4.0 [CTCAE ver.4.0]), pemigatinib was reduced to 9.0 mg/day 3 months after initiating pemigatinib (Figure 2). Nail disorders were managed by reducing the pemigatinib dosage, administering topical steroids and moisturizers, and taping in collaboration with the dermatologist. Hyperphosphatemia was treated with a phosphorus‐restricted diet and lanthanum carbonate. Nutritionists were asked to address taste disorders and recommended that the patient should eat foods that could be tasted easily and gargled with warm water. Due to nail disorders and taste disorders, the pemigatinib dosage was reduced to 4.5 mg/day 2 months after reducing it to 9.0 mg/day. Despite her recurrent cholangitis and strong pemigatinib side effects, she managed to continue pemigatinib with repeated medication withdrawal as needed; however, she died 26 months after being diagnosed with stage IVB ICC. The treatment progression is shown in Figure 3.

## 3 DISCUSSION

We reported a patient with Stage IVB ICC who was treated with pemigatinib. Liquid CGP was useful for detecting FGFR2 gene fusion. Furthermore, the patient experienced typical pemigatinib side effects requiring treatment; however, long‐term survival could still be achieved by multidisciplinary management of side effects. CGP has been approved and used in clinical practice. CGP can detect gene mutation and may verify alternative treatment options. There are currently limited anticancer drugs available for ICC, and some cases may not be treated adequately. Therefore, although CGP has high costs, CGP can identify alternative treatment options and bring significant benefits in these cases. When conducting CGP, detection rates of mutations in the tissue CGP were higher than those in the liquid CGP.^13^ However, previous anticancer treatment may have caused tumor heterogeneity and posttreatment specimen collection may not provide adequate specimens for CGP.^14^ Conversely, liquid CGP can detect gene mutation regardless of tumor heterogeneity and may be more useful as the CGP test than tissue CGP in some cases.^15^ In this case, liver tumor and lymph node samples were inappropriate for tissue CGP; however, liquid CGP was useful for detecting gene mutation and FGFR2 fusion. Therefore, tumor and time heterogeneity should be considered in performing CGP, and an appropriate method should be used.^16^, ^17^ New drugs targeting genetic mutations in ICC, such as entrectinib, neurotrophic tyrosine receptor kinase inhibitor or ivosidenib, and isocitrate dehydrogenase 1 inhibitor, have also been approved in recent years.^18^, ^19^ Genetic mutations may change during treatment. Therefore, accurate timing and frequency of performing CGP should also be verified in the future.^16^, ^17^ Reports on the use of pemigatinib for ICC in the real‐world setting are limited. Pemigatinib has characteristic side effects, for example, nail disorders, taste disorders, and hyperphosphatemia as in this case. The pemigatinib doses should be reduced due to severe side effects, such as nail and taste disorders. Side effects of pemigatinib can be managed by reducing the dosage,^20^ and in this case, reducing the dosage or medication withdrawal was effective in management of the side effects. Genetic factors are known to affect the occurrence of side effects in other anticancer drugs,^21^ but genetic factors associated with side effects in pemigatinib are not clear. Therefore, the influence of genetic factors on the occurrence of side effects of FGFR inhibitors should be investigated in the future. However, the patient continued the pemigatinib treatment through multidisciplinary treatment. Newly developed anticancer drugs may cause different side effects from conventional anticancer drugs. In this case, side effects required a dose reduction; however, multidisciplinary management enabled the patient to continue treatment, which achieved a long‐term survival of approximately 26 months. Thus, a multidisciplinary team that can contribute to a good response and prolonged survival should be established.

In conclusion, we managed a patient treated with pemigatinib. Liquid CGP is useful for detecting FGFR2 fusion. Pemigatinib caused typical side effects, including nail disorders, hyperphosphatemia, and taste disorders; however, multidisciplinary treatment enabled us to continue the treatment and achieve long‐term survival.

## AUTHOR CONTRIBUTIONS

**Shun Ishido:** Conceptualization; data curation; writing – original draft. **Nobuharu Tamaki:** Conceptualization; data curation; writing – original draft. **Kento Inada:** Data curation. **Jun Itakura:** Data curation. **Yuka Takahashi:** Data curation. **Naoki Uchihara:** Data curation. **Keito Suzuki:** Data curation. **Yuki Tanaka:** Data curation. **Haruka Miyamoto:** Data curation. **Michiko Yamada:** Data curation. **Hiroaki Matsumoto:** Data curation. **Tsubasa Nobusawa:** Data curation. **Taisei Keitoku:** Data curation. **Kenta Takaura:** Data curation. **Shohei Tanaka:** Data curation. **Chiaki Maeyashiki:** Data curation. **Yutaka Yasui:** Data curation. **Kaoru Tsuchiya:** Data curation. **Hiroyuki Nakanishi:** Data curation. **Masayuki kurosaki:** Funding acquisition; supervision; writing – review and editing. **Namiki Izumi:** Supervision; writing – review and editing.

## CONFLICT OF INTEREST STATEMENT

All authors have no conflicts of interest to declare.

## CONSENT

We obtained written informed consent from patient to publish this report in accordance with the journal's patient consent policy.

## Data Availability

The datasets are not publicly available (because the datasets have personal information) but are available from the corresponding author on reasonable request.
